# The effect of active occupational stress management on psychosocial and physiological wellbeing: a pilot study

**DOI:** 10.1186/s12911-020-01347-z

**Published:** 2020-12-03

**Authors:** Tomislav Jukic, Alojz Ihan, Vojko Strojnik, David Stubljar, Andrej Starc

**Affiliations:** 1 Department of Internal medicine, Family medicine and History of Medicine, Faculty of Medicine Josip Juraj Strossmayer, Osijek, Croatia; 2 Institute of Microbiology and Immunology, Medical Faculty of Ljubljana, Ljubljana, Slovenia; 3 Faculty of Sport, University of Ljubljana, Ljubljana, Slovenia; 4 Department of Research & Development, In-Medico, Mestni trg 11, 8330 Metlika, Slovenia; 5 Chair of Public Health, Faculty of Health Sciences, University of Ljubljana, Ljubljana, Slovenia

**Keywords:** 24alife, E-tools, Healthcare applications, Occupational health, Prevention

## Abstract

**Background:**

The aim of the study was to address the working population with an occupational stress prevention program using mHealth solution and encourage them for healthy lifestyle choices.

**Methods:**

Seventeen participants were randomized from the corporate setting. A 24alife app with a good compliance program was selected. Test battery has been designed to test the physical readiness, psychological evaluation and biological blood markers for stress. Participants were followed up after 30, 60 and 90 days, respectively, within the intervention period. Weight of participants was tracked three times per month. Univariate analysis compared the continuous variables by One-Way Repeated-Measures ANOVA test when the data were normally distributed, or Wilcoxon rank sum test for abnormal distribution of variables.

**Results:**

Participants used the app with a compliance rate of 94.1%. The psychological evaluation revealed higher motivation for work, lower burnout scores and participants gave subjective responses of better general wellbeing. Some of the participants lost up to four kg of body mass. Physical readiness has also improved.

**Conclusions:**

Results of mHealth projects on corporate could include primary health care institutions and health ministry to extend the existing system to patients’ pockets where they can monitor their disease and increase the ability of self-care.

## Background

The psychological and physiological effects of stress in the workplace have been established as the risk factors for mental and physical health problems [1]. Along with the mobile applications, that make our life easier, the trend in the past few years focused on solutions that aim the most personal value of an individual – health [2, 3]. Psychological responses to stress are often associated with negative emotions. Cognitive processes, such as rumination and worry, can prolong stress responses. Such recurring activation of cognitive representation of stress, called preservative cognition, can induce physiological activation of stress-response systems [4]. Those cognitive processes are considered to act as psychological responses to prolonged stress and are usually indicated by negative emotions such as anxiety, depression and anger [5]. Occupational strain, has been shown to lead to general decrease in wellbeing, psychological strain with increased anxiety and depression, and lessen cognitive abilities, such as concentration and productivity at work, along with increase in biological stress markers [6–8]. It has been shown that physical activities, proper nutrition adaptation and psychological relaxation techniques, along with social interaction and support, lead to a better resilience to stress [9].

Recently, 24alife [10], an application for healthy lifestyle, has been developed to reach different target groups – elderly, employees, fitness centres and employers. It offers information technology (IT) guided health care promotion program consisting of software for human resources (HR) management and separate, but linked, mobile app and portal-based guidance for employees to manage their stress at work more efficiently. The general idea behind 24alife is that when you use the app and internet portal, it records your activities (how often you do sports, what kind of exercises you prefer), food intake, stress level, weight, blood pressure and other body parameters. It serves as data record and enables you and your doctor to have a more detailed view on your health history. The user can choose a guided program when they need an extra support. Programs are designed to reach desired goal or to improve a certain part of user lifestyle. Fit & Active goal is intended for those, who want to shape their body and stay fit with regular exercise, Relaxed & Confident goal is intended for those, who want to successfully cope with the stressful lifestyle, Diet & Habit goal is intended for those, who want to change their diet habits and preserve their ideal weight with the support of psychological and sport exercises [10].

Current study was designed to address the highly stressed working population with occupational stress prevention program encouraging healthy lifestyle choices that combine the fields of medicine, kinesiology, psychology and nutrition. The automated program was individually adapted and designed to lower stress, increase productivity, decrease absenteeism and increase motivation on the workplace. The aim of this study was to offer and test the intervention program and behavioural changing pattern in employees, who experienced high levels of strain at workplace. Participants were tested for psychological and physiological parameters prior intervention, and were followed up after 30, 60 and 90 days, respectively, within the intervention period. With the preliminary analysis, we aim to test for the optimal time period of an intervention program and gather the data on the psychological and behavioural changes at each time point to reach a conclusion on the most appropriate time frame of the intervention.

## Methods

### Subjects

The participants were selected from the Slovenian corporate setting. Randomization was done from the list of all employees from a company RC IKTS Žalec LLC (limited liability company). Every fourth person from the list of employees was selected for the study inclusion. The participants were given written informed consent form and research information sheet prior the inclusion. Seventeen participants, 9 males and 8 females, decided to undergo the testing. One male participant was excluded from the study due to inability to finish the program with the final testing after 90 days. The mean age of participants was 34.9 (SD = 10.1) years. Personal details such as marital status, children were not obtained as they were not relevant for the objectives of the analysis. No investigator had access to the participants’ health screen or medical records to ensure confidentiality. Under the principles of protection of human subjects, no information was gathered on individuals, who declined participation. The study procedure was conducted according to the Declaration of Helsinki and approved by the departmental committee. Participants gave written consent of participation.

### Test battery

Test battery was designed to test the physical readiness, psychological evaluation and to assess biological blood markers for stress. The participants underwent testing on the first day of the intervention and then 30, 60 and 90 days after the start of the intervention.

### Physical readiness evaluation

Participants’ cardiovascular fitness levels were measured using the Rockport Fitness Walking Test [11]. Participants’ level of flexibility was measured with a standardized Sit and Reach Test. Level of muscular strength was measured by the Sit up test.

### Rockport test (Vo2max test)

Participants’ cardiovascular fitness levels were measured using the Rockport Fitness Walking Test (RFWT) [11]. The RFWT is a 1600 m (m) walk test in which participants are instructed to walk the distance as quickly as possible. Results of several studies support the use of the RFWT as a reliable and valid instrument for healthy or adults with intellectual disabilities (ID). The test-retest reliability of the RFWT is high (r = 0.99) [11]. Each participant underwent an aerobic power testing at their first meeting.

### Muscle flexibility test

Participant’s level of flexibility was measured with a standardized Sit and Reach Test. The test measures flexibility of the lower back and hamstrings. It requires a box of about 30 cm width and height. The participants are required to sit on the floor with legs straight ahead and knees flat against the floor. Feet should be placed flat against the box, leaning forward slowly as far as possible keeping the fingertips level with each other and the legs flat reaching forward towards the box. The furthest point to where participants can reach after a second attempt is marked as the flexibility level based on the standardized categories.

### Muscle strength test

Level of muscular strength was measured by the Sit up test, where the participants were required to follow a tempo of 20 per minute. Abdominal muscle strength and endurance is important for core stability and back support. This sit up test measures the strength and endurance of the abdominals and hip-flexor muscles. The participants were lying on a cushioned floor with their knees bent at approximately right angles, with feet flat on the ground. Hands were resting on their thighs. The participants were required to squeeze the stomach, push their back flat and raise high enough for the hands to slide along the thighs to touch the tops of the knees. Then they returned to the starting position and repeated the steps.

### Psychological evaluation

24alife assesses stress profile with the help of psychological questionnaires. The following questionnaires were used:

*General Health Questionnaire* (GHQ) is a 12-item version or GHQ-12 [12, 13], a self-reported instrument of psychological components of health. The GHQ-12 focuses on breaks in normal function (rather than upon lifelong traits) and concerns itself with two major classes of phenomena: inability to continue to carry out one’s normal “healthy” functions and the appearance of new phenomena of a distressing nature.

*Modified Fatigue Impact Scale* (MFIS) [14] is a well validated 21-item questionnaire assessing several aspects of fatigue and activity. The scale was developed for clinical population particularly for the patients with multiple sclerosis, however it was used in a healthy population for research purposes. Higher scores indicate a higher degree of fatigue. The scale can be interpreted in subscales of physical fatigue, cognitive fatigue, psychosocial fatigue, and the overall score of the fatigue scale. The MFIS contains 9 “physical” items, 10 “cognitive” items, and 2 “psychosocial” items. The maximum possible score is 84, with higher scores indicating a greater impact on quality of life.

*State-Trait Anxiety Inventory* (STAI – X) measures anxiety as a stable personality trait, a persons’ disposition to be nervous instead of the more prominent use of the term assessing an emotional state characterized by subjective feelings of tension, apprehension, nervousness and worry, and by activation or arousal of the autonomic nervous system [15, 16]. Form X of the STAI contains 20 state anxiety items and 20 trait anxiety items. The state anxiety items are each rated on a 4-point intensity scale, from 1 for “not at all” to 4 for “very much so.” The trait anxiety items are rated on a 4-point frequency scale (from “almost never” to “almost always”). Respondents are asked to indicate how they generally feel. Scoring is reversed for anxiety-absent items (e.g., “I feel calm”). STAI was developed as a unidimensional self-report measure. 10 items are positively worded, and 10 items are negatively worded. Score range is 20–80 and higher scores indicate greater levels of anxiety.

*Satisfaction with Life Scale* (SWLS) [17]. The satisfaction with life was obtained by assessing global cognitive judgment of participants’ view of their life on a 5-item scale. There is a 7-point scale from “strongly disagree” to “strongly agree” and a score range is 5–35. A score of 20 represents the neutral point on the scale. Scores between 31 and 35 indicate extremely satisfied, 26–30 indicates satisfied, 21–25 indicates slightly satisfied, 15–19 indicates slightly dissatisfied, 10–14 dissatisfied, and 5–9 extremely dissatisfied. The scale has strong internal reliability and moderate temporal stability.

### Maslow burnout inventory (MBI)

The MBI was created in 1996 by Maslach, Jackson and Leiter [18]. The MBI is the most well-known measure of burnout and has been used in more than 90% of empirical studies on the subject [19, 20]. The three main components of burnout include: emotional exhaustion, depersonalization and personal accomplishment. Each of these three scores is measured using questions answered with a 7-point frequency scale and the answers range from 0 (“never”) to 6 (“everyday”). “Depersonalization” occurs when a teacher isolates himself from others. This variable is measured with five items on the survey that ask for the frequency with which they experience negative feelings towards other teachers and administrators. “Personal accomplishment” is the self-evaluation of the efficacy of the teacher’s own work. Eight items on the survey test the teacher’s feelings of personal accomplishment. “Emotional exhaustion” measures fatigue, frustration, and stress. Nine questions on the survey are used to create a score for this component. The sum of each of the 22 questions yielded a burnout score for individual participants.

### Biological stress marker evaluation

Biological stress markers were repeatedly evaluated. Blood tests of biochemistry and hormonal levels were measured at the beginning and at the end of evaluation. Fasting blood samples (ethylenediaminetetraacetic acid - EDTA tubes, 3 mL) were collected during the morning hours; plasma and cell fractions were processed on the same day of collection. Plasma aliquots were stored at − 80 °C until use.

### Intervention

#### Automated software program

The participants were asked to actively participate and follow the automated software-based program (24alife app), which was designed to holistically approach forming healthy lifestyle habits. The intervention program was a mobile app guide with daily reminders and tasks, designed to guide individual through healthy lifestyle for 3 months. Based on user’s initial state, which was specified testing battery, the algorithm behind the software determined the intensity of the automated program. Each participant got a personalized ratio of psychological relaxation exercises, sports training exercises (endurance training, interval training, strength training), nutrition advice and reminders (eating diary, reminder to drink enough fluids), and reminders to track their bodily response to stress. After initial data input, such as age, height, weight, waist and neck measurements, the software calculated body mass index (BMI), fat and muscle index. The technology ensured that the physical training was safe by tracking the HR zone with alerts on how to adjust the tempo of training.

#### Personalized motivation

Alongside the automated solution, the participants attended four thematic workshops led by field experts. Workshops entitled “Coping with stress”, “Safe exercising”, “Do not feed stress” and “Motivation to keep healthy habits” were organized at the end of each month to educate subjects about stress and motivate participants to be more aware of the negative consequences of stress. Every week the employees joined one-hour group workout led by sports professional. The participants were asked for a feedback on satisfaction with the automated program every 2 weeks.

### Statistical analysis

Statistical analysis was performed using SPSS 21 software (IBM, New York, USA). Univariate analysis compared the continuous variables by One-Way Repeated-Measures analysis of variance (ANOVA) test in case of statistically significant difference for the data that were normally distributed, or Wilcoxon rank sum test for abnormal distribution of variables. Biological markers were measured only twice during the study; thus, One-Way ANOVA was used to compare the values between the measurement on day 1 and measurement after 90 days. Statistical differences were considered to be significant at *P* value < 0.05.

## Results

The results showed that participants frequently used the 24alife app with good compliance to the program. The compliance rate was 94.1% including the subject, who could not perform the tests till the end. Participants tracked their weight three times per month as suggested. They followed 90 min of guided sport exercises for aerobic and strength training per week and listened to 7 min of relaxation exercises per day.

The psychological evaluation revealed higher motivation for work, lower burnout scores and participants gave subjective response of better general wellbeing (Table 1). Participants felt less fatigue and anxiety after 90 days. Psychological evaluations showed the improvement during the 90-day follow-up period, but the differences were not statistically significant. Moreover, participants found the experience enhancing for their teamwork as they compared the results among each other. Additionally, they spontaneously organized group activities in their free time without the knowledge of primary investigators.

**Table 1:** Means and standard deviations of psychological assessments

	General Health (GHQ)	Fatigue (MFIS)	Anxiety (STAI-X)	Satisfaction with life (SWLS)	Burnout total (MBI)
Day 1	53.4 ± 12.6	57.7 ± 5.3	46.4 ± 6.0	25.2 ± 1.0	93.3 ± 10.0
Day 30	54.6 ± 10.0	58.8 ± 18.3	45.1 ± 5.0	26.8 ± 4.1	92.1 ± 12.0
Day 60	58.8 ± 18.3	54.6 ± 10.0	43.8 ± 5.0	26.5 ± 5.6	91.1 ± 11.0
Day 90	57.7 ± 5.3	53.4 ± 12.6	42.9 ± 6.3	27.2 ± 5.3	89.9 ± 14.0

On average, body measurements have not improved much (differences between day 1 and day 90 were not statistically significant), however, on the individual level, some of the participants lost up to four kg of body mass. There was a trend of a positive change by the end of the program in decrease of body weight, body fat and BMI (Table 2) and improvement of parameters for physical readiness (Table 3). Most changes were observed after 90 days, but ANOVA did not show any statistical significance throughout the time periods (Table 3).

**Table 2:** Means and standard deviations of body measurements

	Weight [kg]	Fat [%]	Muscle mass [%]	BMI
Day 1	69.8 ± 13.7	26.4 ± 5.8	32.6 ± 4.4	23.5 ± 3.4
Day 30	69.3 ± 13.1	25.0 ± 6.5	33.8 ± 4.8	23.3 ± 2.6
Day 60	69.1 ± 13.0	24.9 ± 6.2	33.8 ± 4.6	23.2 ± 2.5
Day 90	68.5 ± 12.6	23.8 ± 5.2	34.8 ± 4.1	22.9 ± 2.5

**Table 3:** Means and standard deviations of physical readiness

	Strength	Flexibility	Rockport time	Rockport pulse	Vo2 max
Day 1	50.4 ± 19.2	3.5 ± 1.0	13:33 ± 0:50	141.1 ± 19.0	44.5 ± 13.2
Day 30	54.7 ± 10.7	3.6 ± 0.8	12:43 ± 01:06	156.9 ± 99.8	46.3 ± 14.4
Day 60	55.8 ± 8.7	3.6 ± 0.8	12:51 ± 01:18	144.3 ± 20.8	47.4 ± 6.6
Day 90	56.2 ± 7.4	3.6 ± 1.2	12:40 ± 01:48	156.8 ± 20.3	45.2 ± 3.9

Moreover, the most evident positive results were seen in the biological markers of stress (Table 4). A significant decrease in stress hormone cortisol, C-reactive protein (CRP) and glucose level by the end of the program was observed with simple One-Way ANOVA test (Table 4).

**Table 4:** Biological stress markers on day 1 and day 90

	Day 1	Day 90	Value	Ref. Value	*p*-value
Leucocytes	6.9	6.7	10^9	4.0–10.0	0.612
Erythrocytes	5.1	5.1	10^12	4.2–5.4	0.549
Haemoglobin	155.2	156.0	g/L	120–160	0.331
Neutrophils	3.9	3.8	10^9/L	1.6–7.5	0.280
Lymphocytes	5.4	5.7	10^9/L	0.8–5.0	0.349
Monocytes	0.6	0.6	10^9/L	0.1–1.0	0.597
Glucose	4.7	4.5	mmol/L	3.6–6.1	0.042**
CK	2.1	1.8	μkat/L	< 2.85	0.297
CRP	2.6	0.9	mg/L	< 5	0.021**
Cholesterol	4.9	4.9	mmol/L	4.0–5.2	0.292
HDL	1.5	1.4	mmol/L	> 1.4	0.292
LDL	3.2	2.9	mmol/L	2.0–3.5	0.612
Triglycerides	1.1	1.0	mmol/L	0.6–1.7	0.391
TSH	2.8	2.3	mlU/L	0.3–4.2	0.247
Cortisol	603.5	437.1	nmol/L	2.8–8.0	0.012**
Testosterone	4.9	5.4	μg/L	171–536	0.423

*CK *C*reatine kinase, CRP C-reactive protein, HDL *High* density lipoprotein, LDL *L*ow density lipoprotein, TSH *T*hyroid-stimulating hormone

**Statistically significant difference between the values at day 1 compared to day 90 according to One-Way ANOVA test

## Discussion

Mobile applications have been developed to collect physiological, psychological and performance data, however the reliability and validity of such data are often unknown [21]. An evaluation of such apps is warranted and necessary, as according to the review by Buechi et al. [22] there were more than 165,000 available applications to download and not all were showing reliable data process. Whilst mobile apps may have the potential to collect data, athletes and practitioners should take caution when implementing them into practice. 24alife not only handles the standard physiological and psychological tests, but also supports medical data and correlates with the blood markers.

Previous reports confirmed the efficacy, effectiveness and usability of mHealth apps as they managed to change users’ behaviour [23–27], lower depression at workplace [28] at low-costs [29] Apps even proved to improve quality of life and psychological state of patients with chronic diseases [30, 31]. However, barriers were identified in a way of using apps, namely clinicians or caregivers were not ready to use them [32]. Research with diagnostic studies are missing since most apps target a diagnostic problem without the scientific basis [22]. Our study is a good example of how a mHealth solution might be integrated into the occupational health programs (OHP) and prevention programs. The results showed significant changes in levels of stress hormones which could be due to increased level of physical activity and frequency of deep relaxation during a regular week for employees. The significant decrease of the glucose level could be explained by the lower level of stress and daily reminders on the mobile phone app to start exercising or exercising more. Another reason is also, that endurance training recognizes the input from the brain, including an ability to cope with various non-pleasurable perceptions during exercise, such as pain and temperature. Exercise training can reduce perceptions of pain and temperature over time [33], so the participants could feel more relaxed in every-day situations.

Similar was observed in the study by Stuckey et al. [34] where the authors investigated the effects of an exercise prescription supported by mHealth app compared to an exercise prescription alone to improve metabolic syndrome and cardiometabolic risk factors. The primary outcome was that systolic blood pressure was greatly reduced in the mHealth-supported intervention group at 12-weeks follow-up and this improvement was better maintained for the whole trial year. In general, exercise has positive health benefits. The effects of exercise on individual metabolic syndrome risk factors have been previously extensively reviewed [35–37]. Training interventions were 8 to 52 weeks, frequency of 2 to 5 sessions per week for 40–60 min per session at moderate to high intensity. Endurance exercise training reduced waist circumference, lowered systolic blood pressure and diastolic blood pressure and increased high density lipoprotein cholesterol [38]. Additionally, lifestyle interventions have also shown to improve autonomic [39–42] and vascular function as well [43, 44]. Importantly, improvements in heart rate variability with lifestyle interventions were associated with lower risk of diabetes, independent of weight loss and physical activity [45]. Moreover, recent study by Fuller-Tyszkiewicz et al. [46] evaluated very similar app and confirmed these findings. The authors proved that mHealth psychological interventions present an effective treatment option for users since the intervention group experienced significant reductions in stress and depressive symptoms, and higher levels of emotional well-being.

Use of mHealth tools for fitness assessment and exercise prescription has been observed to improve the general health and feeling of subjects [47]. Increased cardiorespiratory fitness was shown to reduce mortality due to cardiovascular events in men [48]. Intensive lifestyle intervention programs reduced the risk of developing type 2 diabetes mellitus in patients with prediabetes and improvements were maintained over a long-term follow-up [49]. All these findings and the results of our preliminary study highlighted the importance of post-program support for long-term maintenance. Using mHealth apps improves participants/users’ health and benefits in reducing work strain. Implementation of such activities is important to translate research protocols into useable programs including the construction of a national network of partners with interest in chronic disease management. The digitization of records and network creation might strengthen monitoring [50]. Identified disadvantages of mHealth apps are mostly technological, lack of face-to-face contact, training of users and resources [51].

Our study showed positive changes towards healthier lifestyle habits with better physical and psychological well-being, and even found lower levels of perceived and biological stress among participants. However, we need the follow-up study which will be able to reveal the factors that are necessary for the e-solution to be successful in health changes. This study had strengths and limitations. This study assessing the performance of diagnostic health app using smartphone showed that scientific evidence is necessary. Our study design was good, and the major strength was the detailed preparatory work which was undertaken to develop the application and specifically adapt it into corporate. Another strength is the subsequent evaluation and usability indicators of the monitoring system. This is one of the few apps currently available on app stores that include assessed parameters of diagnostic accuracy. A limitation is that the pilot study period was relatively short, and it is therefore not possible to distinguish long-term results. It only showed preliminary results and is subjected to many limitations preventing us to draw conclusions on the efficacy of the automated program. Despite a pilot study, a small sample size of only 16 participants was used. Therefore, the findings need to be interpreted with some caution. A larger study sample including athletes, semi-professional athletes, and more employees may also have provided further insights into the feasibility and usability of this relatively novel mHealth application. Participants volunteered to be enrolled in the study. Based on the inclusion criteria, participants were exposed to the occupational stress, and thus the population consisted of highly motivated individuals, who were interested in improvements of their lifestyle. Therefore, the findings may not be generalizable to a general population, as many at-risk individuals are not motivated to change their behaviour.

## Conclusion

In health issues, where changing lifestyle is one of the crucial factors for slowing the progress of chronic disease such as stress, burnout or diabetes, patients could benefit from holistic personal monitor such as 24alife. We showed that preliminary results of mHealth projects on corporate could include primary health care institutions and health ministry to extend the existing system to patients’ pockets where they can monitor their disease and increase the ability of self-care. Based on the results from a healthy population we could create a tool that will ease the patients’ transition from the first diagnose to self-care, and most importantly create a solution where the management of chronic illness will be more successful, patients will be able to change their lifestyle and, by the help of support and reminders, form healthier habits with long-term positive consequences to their health.

## Data Availability

The data that support the findings of this study are available from the company RC IKTS Žalec but restrictions apply to the availability of these data, which were used under license for the current study, and so are not publicly available. Data are however available from the authors upon reasonable request and with permission of the company RC IKTS Žalec.

## Abbreviations

ANOVA: Analysis of variance
BMI: Body mass index
CRP: C-reactive protein
EDTA: Ethylenediaminetetraacetic acid
GHQ: General Health Questionnaire
HR: Human resources
ID: Intellectual disabilities
IT: Information technology
LLC: Limited Liability Company
m: Meter
MBI: Maslow Burnout Inventory
MFIS: Modified Fatigue Impact Scale
OH: Occupational health programs
RFWT: Rockport Fitness Walking Test
SD: Standard deviation
STAI: State-Trait Anxiety Inventory
SWLS: Satisfaction with Life Scale
